# Non-interferometric phase retrieval using refractive index manipulation

**DOI:** 10.1038/srep46223

**Published:** 2017-04-07

**Authors:** Chyong-Hua Chen, Hsin-Feng Hsu, Hou-Ren Chen, Wen-Feng Hsieh

**Affiliations:** 1 Department of Photonics, National Chiao Tung University, Hsinchu 300, Taiwan

## Abstract

We present a novel, inexpensive and non-interferometric technique to retrieve phase images by using a liquid crystal phase shifter without including any physically moving parts. First, we derive a new equation of the intensity-phase relation with respect to the change of refractive index, which is similar to the transport of the intensity equation. The equation indicates that this technique is unneeded to consider the variation of magnifications between optical images. For proof of the concept, we use a liquid crystal mixture MLC 2144 to manufacture a phase shifter and to capture the optical images in a rapid succession by electrically tuning the applied voltage of the phase shifter. Experimental results demonstrate that this technique is capable of reconstructing high-resolution phase images and to realize the thickness profile of a microlens array quantitatively.

Phase imaging is important and attractive in wide applications^1^,^2^, such as phase contrast optical microscopy for biological studies of cells^3^ and electron microscopy, because many samples are highly transparent to the light beam and electron beam but exhibit strong phase contrast. The phase images could provide better resolution than the intensity images and some might have their imaging resolution beyond the resolution of Rayleigh’s criterion^4^. Conventionally, the phase images are measured by the interferometry technologies that stringently require the temporal and spatial coherence of the optical sources^5^,^6^,^7^,^8^,^9^. On the contrary, the technique based on the transport-of-intensity equation (TIE) which can deduce the phase image only from several intensity measurements along the propagation direction is a simple and non-interferometric method and less sensitive to coherence in the illumination^10^,^11^. However, precise control of physical moving parts to translate the camera or the object mechanically is required to acquire a series of through-focus images for phase reconstruction^11^,^12^,^13^. This mechanical movement gives rise to a complication of the acquisition process and the significant acquisition time.

Recently, several schemes, such as volume holography^14^, chromatic aberrations^15^, spatial light modulator^16^,^17^,^18^,^19^, flow cytometry^20^ and electrically tuning lens^21^, were presented to avoid the mechanical motion. Most of these approaches require a 4-f configuration into the acquisition systems. However, variations in magnifications and the locations between images are needed to compute precisely to achieve better phase images in these methods. Additionally, these systems become complicated, and the alignment would be crucial to realize the best resolved images. Although a flow-cytometry scheme uses a microfluid device to translate the object through the focal plane of a microscope, it is only available for the non-adherent cells.

In this paper, we present a novel and simple imaging acquisition system with no moving elements by inserting an electrically-tuned liquid crystal (LC) phase shifter in between the specimen and the camera to acquire the intensity image stacks. Base on this system, we obtain an equation for the intensity-phase relation with respect to the variation of refractive index which is similar to the TIE. From a mathematical point of the view, we can use the available TIE solvers to calculate the phase images without the need to take into account of the variations in magnifications and locations among the optical images. Subsequently, an in-line transmissive imaging system where a microscope lens magnifies the objects onto a CCD camera placed at the output image plane is used to demonstrate the concepts. Different images are recorded by electrically tuning the extraordinary refractive index of the phase shifter. Once the image stacks are stored in the computer, they are numerically computed in a similar way presented in Ref. 22, and the phase information of the objects is quantitatively recovered.

## Method

### Theoretical analysis

Consider a monochromatic incident beam propagating along the z axis. After passing through an object, the incident wave transmits a medium with a thickness of L and a tunable refractive index. Under the paraxial approximation, the complex field after passing through the object is


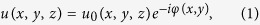


where *u*_0_(*x, y, z*) is a slowly varying field amplitude and φ(x, y) carries the phase distribution of the object, x and y represent the positions transverse to the z (optic) axis. Let the corresponding angular spectrum of u_0_(x, y, z) be U_0_(k_x_, k_y_, z) with the angular frequencies k_x_ and k_y_. As this beam propagates through the medium with the refractive index of n, the resultant angular spectrum becomes


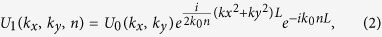


where k_0_ is the wavenumber.

Whereas, this beam propagates through this medium having its refractive index tuned to n + Δn, the resultant angular spectrum is


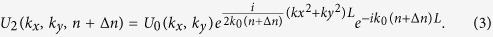


Then, the rate of change of the angular spectrum with respect to the refractive index is


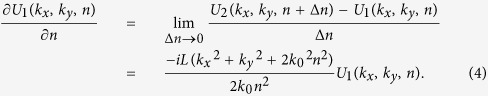


By applying the inverse Fourier transform of (4), we obtain the differential equation of


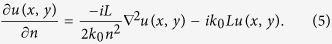


where ∇^2^ represents the 2D Laplacian operator over x and y.

We observe that Eq. (5) has a similar form of the paraxial wave equation. Thus, we can derive an equation, similar to the TIE, to describe the intensity change due to the refractive index variation in the medium. Let 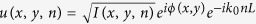 with the real-valued intensity distribution I(x, y, n) and spatially-varying phase ϕ(x, y) and substitute into Eq. (5), we obtain the new intensity-phase equation


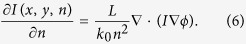


This equation states that the phase information can be retrieved in the variation of the intensity with respect to the refractive index change. An estimate of the intensity derivative with respect to n can be obtained by measuring different intensity images at a sufficiently close variation of the refractive index. As a result, we propose a practical approach to electrically tune the extraordinary refractive index of the LC by using a LC phase shifter as the medium. After acquiring a series of intensity image stacks with different applied voltages, the phase ϕ(x, y) is quantitatively computed by solving this new intensity-phase equation numerically based on a TIE solver by using Hilbert transform technique or by using fast Fourier transform (FFT)^22^, where the derivative of I with respective to n is approximately calculated by using the finite difference (FD) method, i.e., 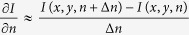.

### Experiments

#### Fabrication of LC phase shifter

**Figure 1:**
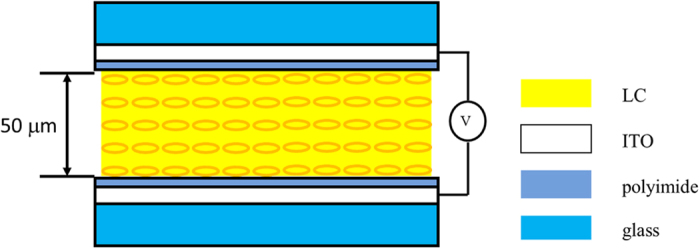
Schematic diagram of the LC phase shifter.

Figure 1 shows the configuration of the LC phase shifter. A LC mixture MLC 2144 (Merck) is injected into an empty cell consisting of two glass substrates where a thin conductive indium-tin-oxide (ITO) layer and the polyimide (PI) layer are coated on the inner surfaces. The homogeneous alignment of the LC layer is obtained by gently rubbing the surface of polyimide layers. The thicknesses of the glass substrates are approximately 700 μm, and that of the LC layer is 50 μm. A thick LC layer is chosen to obtain large enough phase shift because later we would investigate the accuracy of the acquired phase images with respect to the refractive index variation. As a result, the response time in our system is not the issue of the current concern. The ordinary and extraordinary refractive indices of MLC 2144 are 1.5119 and 1.7612, respectively^23^, at the wavelength of 589.3 nm. Before turning on the voltage, the MLC 2144 molecules are parallel to the substrates in an initially stable state.

**Figure 2:**
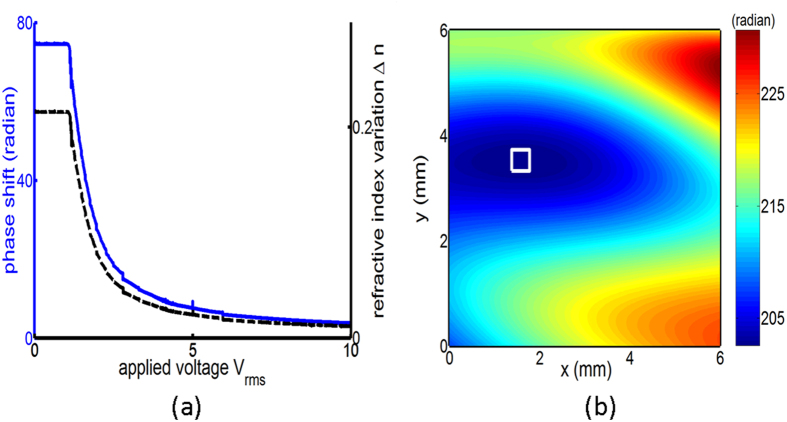
(**a**) The phase shift (blue curve) and corresponding refractive-index variation (black dashed curve) of the LC phase shifter as a function of the applied voltage. (**b**) The measured wavefront phase of the phase shifter with an applied voltage of 2.344 V_rms_. The inset is the region inside which the phase variation is less than 0.2 radians.

The phase shift dependence on the applied voltage is characterized by sandwiching the LC phase shifter between two crossed polarizers at an incident wavelength of 633 nm. The polarizers are rotated at 45° with respect to the axes of ordinary and extraordinary rays. The driven voltage is a square-wave voltage at a frequency of f = 1 kHz. The phase retardation between the ordinary and extraordinary rays is calculated directly from the measured transmission^24^. Then, the calculated phase shift and the corresponding refractive index variation of the extraordinary ray as a function of the applied voltage is shown in Fig. 2(a). We observe that the threshold voltage for LC molecules essentially aligned with the applied electric field is 0.5 V_rms_. The voltage to start saturating the LC response is roughly 1.4 V_rms_. This device has approximately a sharp linear response at the bias voltage within a range between 0.55 V_rms_ and 1.4 V_rms_. By taking into account the precision of the voltage setting and the mapping from the transmittance to the refractive index, the refractive index variation has an accuracy of 10^–3^. The dynamic response of this phase shifter is measured by applying a square voltage with a voltage of 2.344 V_rms_ at f = 1 kHz. The measured rise time is roughly 1 second (sec) and the fall time is 9.2 sec. We can roughly record 5 optical images within a minute by tuning the applied voltage of the phase shifter. Large intensity stack might be realized by replacing LC materials with fast response time, such as blue-phase LCs, or by reducing the cell thickness.

In order to examine the uniformity of refractive index of our fabricated liquid crystal phase shifter, we measured the wavefront phase in use of a wavefront sensor (WFS150-7AR, Thorlabs). The result shown in Fig. 2(b) reveals that a reasonable flat wavefront (having the phase variation less than 0.2 radians) within an area of 400 μm × 400 μm is found, which is larger than the sizes of the concerned specimens that is less than 250 μm × 250 μm. The incident wave impinges upon this region, and then we can assume that the refractive index variation is uniformly distributed for the whole images.

#### Experimental apparatus

**Figure 3:**
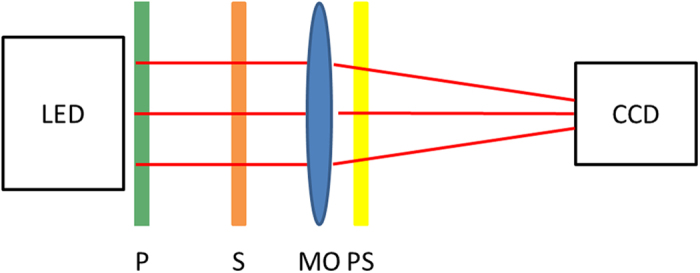
The configuration of the experimental setup: P, polarizer; S, test sample; MO, microscope objective; PS, phase shifter.

The experimental arrangement is exhibited in Fig. 3. A red LED with a low temporal coherence at a wavelength of 633 nm is used to uniformly illuminate the object. The incident LED light is polarized by a polarizer along the axis of the extraordinary ray (e-ray) axis of the LC. A microscope objective (Olympus, MPLAPON 50X, NA = 0.5) placed behind the object is used to focus the image field onto the camera (Nikon-D600) with 6016 × 4016 pixels and pixel pitch sizes of 6 μm. The LC phase shifter is put in between the lens and the camera. The image stack is obtained by tuning the applied voltage of the phase shifter. For measuring a birefringent object, a setup similar to the polarized optical microscope could be used. An analyzer is inserted between the object and the phase shifter and set parallel to the axis of the extraordinary ray. Then, the phase images are measured by rotating the polarizer prior to the object.

## Results

In this section, we provide a discussion of the effect of changing the refractive index on the image quality numerically and measure two test objects, a positive 1951USAF resolution test target (R3L3S1P, Thorlabs) and a microlens array (made by VisEra Co.) as a phase array object, to demonstrate the resolution performance and quantitatively retrieve the phase difference (height) of the microlens array, respectively. In addition, these specimens are also measured by using conventional TIE method for a comparison. In order to suppress noise effect in the conventional TIE method, we use five images to calculate the intensity derivatives by using five-point stencil. On the other hand, we used only two images to calculate intensity derivatives in our proposed approach and the noise was clearly mitigated.

### Effect of the refractive index variation on the image quality

**Figure 4:**
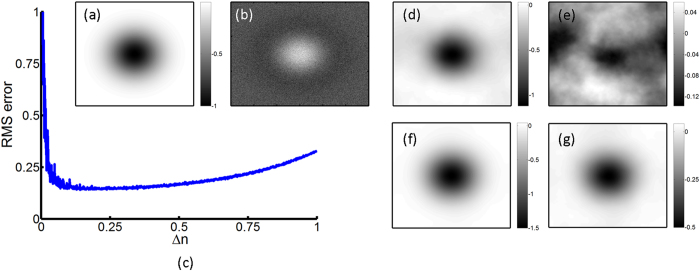
(**a**) The ideal phase distribution, (**b**) the intensity image with noise, (**c**) RMS error as a function of Δn in this TIE imaging, (**d**) phase reconstruction for Δn = 0.2, (**e**) phase error distribution for Δn = 0.2, (**f**) phase reconstruction for Δn = 0.6 and (**g**) phase error distribution for Δn = 0.6.

Equation (6) is similar to the TIE, and we use FD methods to approximate the derivative by using two images. As a result, an optimal choice of Δn is necessary in order to avoid unwanted nonlinearity error and the noisy phase reconstructions^13^,^25^. In simulations, a phase specimen with a Gaussian profile is used, as shown in Fig. 4(a). The incident wavelength is 633 nm and the length of the phase shifter is 50 μm. A 1% white Gaussian noise is added to each intensity image, as shown in Fig. 4(b). Figure 4(c) shows the root-mean-squared (RMS) error in phase construction as a function of Δn. We can see that minimal RMS is obtained as Δn is approximately 0.2. The corresponding optical path difference for this refractive index variation is 10 μm. This optical path difference nearly equals to the optimal Δz for the conventional TIE method using mechanical translation^22^. Furthermore, to have a signal to noise larger than 10, the cell thickness may be further reduced to several μm based on the results in Fig. 2(b).

Figures 4(d) and (e) show the phase reconstruction at the optimal Δn = 0.2 and the corresponding phase error. We can see that the reconstructed phase profile is similar to the phase specimen except that small amount of noise is residual. When Δn increases, the restored phase image become enlarged and blurred due to the error of the FD approximation, as shown in Fig. 4(f) and (g). As a result, in our method there is an optimal Δn to achieve the best phase result.

### Characterization of an USAF target

**Figure 5:**
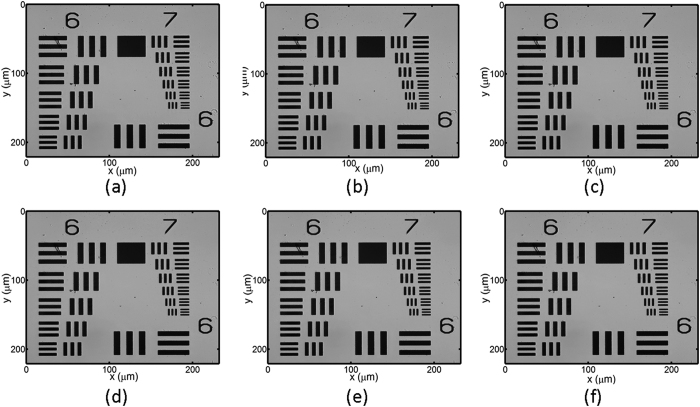
The recorded optical image of the USAF test target with different applied voltages which corresponds to (**a**) Δn = 0, (**b**) Δn = 0.0011, (**c**) Δn = 0.011, (**d**) Δn = 0.0915, (**e**) Δn = 0.184 and (**f**) Δn = 0.205.

**Figure 6:**
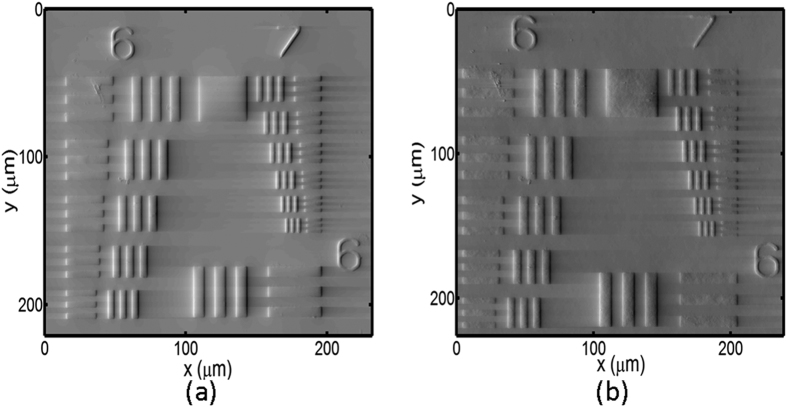
The resolved phase images of the resolution target by (**a**) the current approach with Δn = 0.205 and (**b**) conventional TIE method.

Figure 5 presents the optical image with different applied voltages where the groups 6 and 7 of the USAF target are directly imaged onto the CCD camera. These optical images are approximately the same as the applied voltage is varied mainly due to the small refractive index variation. The smallest lines are in the group 7, element 6 with a line width of 2.19 μm. Furthermore, it shows this target is roughly magnified 100 times, thus the corresponding calculated pixel pitch size is 0.06 μm. Figure 6 displays the retrieved phase images using Eq. (6) with Δn of 0.205 and the conventional TIE method with 10 μm optical path difference. As can be seen in Fig. 6, all lines in both phase images are completely resolved as a result of the Hilbert transform which provides enhanced phase changes with a large phase gradient. That is, the smallest resolvable element is group 7, element 6, defining a lateral resolution of 2.19 μm. In addition, our approach provides a better image quality with better noise suppression compared to the conventional TIE technique. There are some horizontal lines in both images because we apply one-dimensional Hilbert transform horizontally to solve the TIE equation.

**Figure 7:**
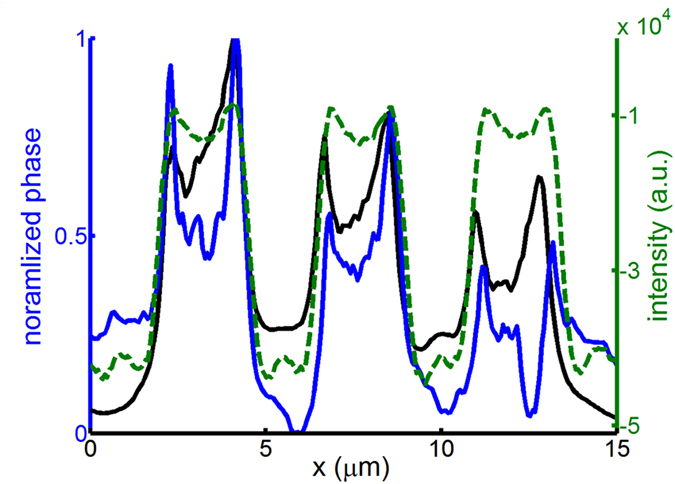
Normalized phase distributions for group 7, element 6 obtained by the current approach with Δn = 0.205 (black curve) and conventional TIE method (blue curve). The dark green dashed curve is the corresponding intensity distribution of the optical image.

Figure 7 exhibits the normalized phase distributions (i.e., each phase distribution is divided by the maximal phase value) for the group 7, element 6 obtained by our proposed approach and the conventional TIE method and the corresponding optical image. The resolved line patterns by using both approaches are in a close agreement with that obtained from the optical image. In addition, the curve by this approach is smoother than that by the conventional TIE method. These results show that we can reproduce the phase image of the USAF target with a comparable resolution successfully by using the LC phase shifter.

### Characterization of a microlens array as a phase object

**Figure 8:**
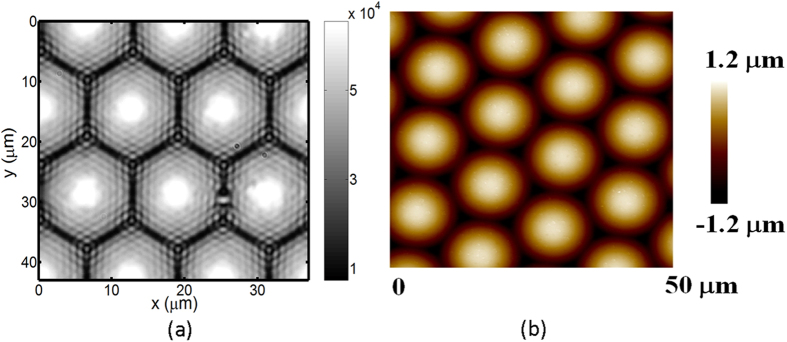
(**a**) The recorded optical image for Δn = 0 and (**b**) the tomography measured by the AFM of the ML array.

**Figure 9:**
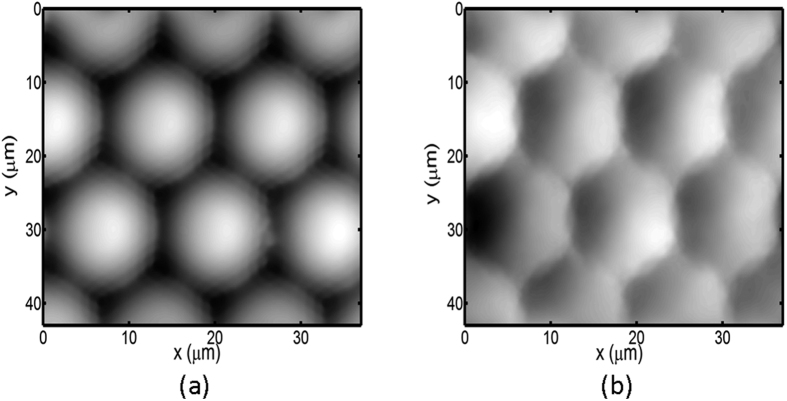
The resolved phase images of the microlens array by (**a**) the current approach with Δn = 0.205 and (**b**) the conventional TIE method.

**Figure 10:**
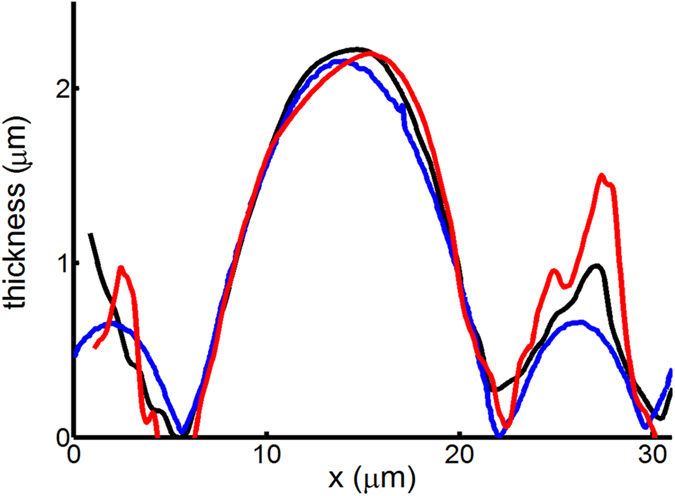
The tomography of the microlens measured by the AFM (blue curve) and the calculated thicknesses of a microlens by the current approach with Δn = 0.205 (black curve) and by the conventional TIE method (red curve).

Figure 8(a) and (b) show the optical image and tomography measured by the atomic force microscope (AFM) of a plano-convex microlens array, respectively. By using the aforementioned pixel pitch size, the calculated pitch of microlens array is roughly 14.31 μm, which is slightly larger than 14.04 μm obtained by AFM. In addition, we observe interference patterns in each microlens element as a result of multiple beam interference. Here, we use the TIE solver by FFT because these interference patterns might result in rippled phase images after Hilbert transform. Figure 9 reveals the retrieved phase images of the microlens array obtained by using Eq. (6) with Δn of 0.205 and the conventional TIE method. We can see that each microlens is clearly resolved without noise fluctuation. Figure 10 illustrates the resolved thickness distribution of the microlens and the tomography measured by the AFM. The resolved profiles closely agree with that measured by the AFM.

## Discussion

In summary, we have demonstrated a new quantitative phase retrieval system by introducing a thick LC phase shifter with an electrically-tuned refractive index between the object and the camera to rapidly record optical images with no moving elements. We carried out a new equation for the intensity-phase relation with respect to the change of refractive index, which is similar to the TIE, and then used the available TIE solvers to recover the phase images without considering the variations of the magnifications and locations between successive optical images. We used a resolution test target and a microlens array as the objects and showed this system can be successfully applied to reproduce the test target image with high resolution and to measure the thickness distributions of the microlens array accurately. Therefore, this method can not only recover the intensity object with high imaging quality but also retrieve the phase object quantitatively. Although the response time of the used LC phase shifter is slow, it can be accelerated by reducing the cell thickness of several μm as the imaging system maintains a signal to noise ratio greater than 10. Furthermore, this imaging system has a great potential to accomplish the phase images of birefringent objects by introducing an analyzer in front of the CCD camera.

## Additional Information

**How to cite this article:** Chen, C.-H. *et al*. Non-interferometric phase retrieval using refractive index manipulation. *Sci. Rep.* **7**, 46223; doi: 10.1038/srep46223 (2017).

**Publisher's note:** Springer Nature remains neutral with regard to jurisdictional claims in published maps and institutional affiliations.
